# Power of grammatical evolution neural networks to detect gene-gene interactions in the presence of error

**DOI:** 10.1186/1756-0500-1-65

**Published:** 2008-08-13

**Authors:** Alison A Motsinger-Reif, Theresa J Fanelli, Anna C Davis, Marylyn D Ritchie

**Affiliations:** 1 Bioinformatics Research Center, Department of Statistics, North Carolina State University, Raleigh, NC 27695, USA; 2 Center for Human Genetics Research, Vanderbilt University Medical Center, Nashville, TN 37232, USA

## Abstract

**Background:**

With the advent of increasingly efficient means to obtain genetic information, a great insurgence of data has resulted, leading to the need for methods for analyzing this data beyond that of traditional parametric statistical approaches. Recently we introduced Grammatical Evolution Neural Network (GENN), a machine-learning approach to detect gene-gene or gene-environment interactions, also known as epistasis, in high dimensional genetic epidemiological data. GENN has been shown to be highly successful in a range of simulated data, but the impact of error common to real data is unknown. In the current study, we examine the power of GENN to detect interesting interactions in the presence of noise due to genotyping error, missing data, phenocopy, and genetic heterogeneity. Additionally, we compare the performance of GENN to that of another computational method – Multifactor Dimensionality Reduction (MDR).

**Findings:**

GENN is extremely robust to missing data and genotyping error. Phenocopy in a dataset reduces the power of both GENN and MDR. GENN is reasonably robust to genetic heterogeneity and find that in some cases GENN has substantially higher power than MDR to detect functional loci in the presence of genetic heterogeneity.

**Conclusion:**

GENN is a promising method to detect gene-gene interaction, even in the presence of common types of error found in real data.

## Findings

### Background

The field of human genetics is currently experiencing an explosion of genetic data, as genotyping technology becomes more inexpensive and accessible. This creates an analytical challenge for genetic epidemiologists. This challenge is exaggerated in the case of complex diseases since the phenotype is likely the result of many genetic and environmental factors[1,2]. The limitations of traditional parametric statistical tools in searching for such interactions motivate the development of novel computational methods[2-4].

Recently, our group proposed a Grammatical Evolution Neural Network (GENN), a machine-learning approach designed to detect gene-gene interactions in the presence or absence of marginal main effects[5,6]. GENN performs both variable selection and statistical modelling without the computational burden of exhaustively searching all possible variable combinations. GENN uses an evolutionary computation algorithm (grammatical evolution) to build neural networks (NN). NN analogize the parallel processing of the human brain, and are used as non-linear statistical data modeling tools to model complex relationships between inputs and outputs or to find patterns in data[7].

GENN has been shown to have high power to detect interactions in a range of empirical studies with both real and simulated data[5,6,8,9]. The performance of GENN has been compared to other NN strategies and found to have significantly improved performance in large datasets[6]. GENN has also been shown to efficiently scale to large datasets[6].

In the current study, we assess the robustness of GENN to several common types of noise. Data originally simulated for Ritchie et al 2003[10] was used to examine the impact of genotyping error (GE), missing data (MS), phenocopy (PC), and genetic heterogeneity (GH) in six different gene-gene interaction models. Additionally, we examine the impact of all possible combinations of these types of noise to detect potentially synergistic effects. Also, we compare the performance of GENN to that of another method designed to detect interactions – Multifactor Dimensionality Reduction (MDR)[11].

### Grammatical Evolution Neural Networks (GENN)

**Figure 1:**
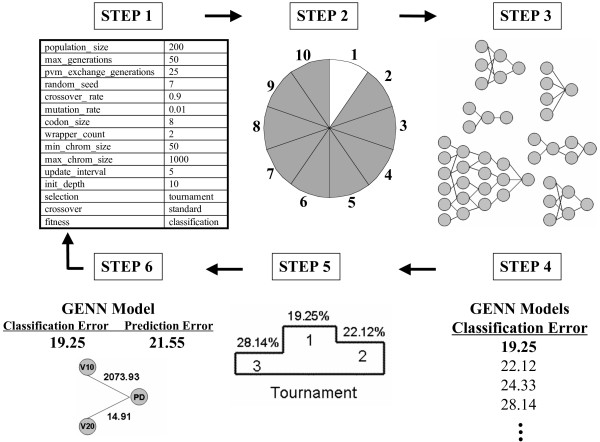
**An overview of the GENN method**. First, a set of parameters must be initialized in the configuration file. These parameters specify details for the evolutionary processes. Second, the data are divided into 10 equal parts for 10-fold cross-validation. Third, training begins by generating an initial population of random solutions using sensible initialization, which guarantees functional NNs in the initial population. Fourth, each newly generated NN is evaluated on the data in the training set and its fitness recorded. Fifth, a selection technique that is specified by the user is used to select the best solutions for crossover and reproduction in the evolutionary process. The cycle begins with the new generation, which is equal in size to the original population. This cycle continues until either a classification error of zero is found or a limit on the number of generations is reached. After each generation, an optimal solution is identified. At the end of GENN evolution, the overall best solution is selected as the optimal NN. Sixth, this best GENN model is tested on the 1/10 of the data left out to estimate the prediction error of the model. Steps two through six are performed ten times with the same parameters settings, each time using a different 9/10 of the data for training and 1/10 of the data for testing. At the end of a GENN analysis, 10 models are generated – one best model from each cross-validation interval. A final model is chosen based on maximization of the cross-validation consistency of variables/loci across the ten models.

GENN methodology and software have been previously described[5,6]. The steps of GENN are shown in Figure 1. Grammatical Evolution is a variation on genetic programming that uses a Backus-Naur Form grammar to create a computer program using a genetic algorithm[12]. A genetic algorithm is an array of bits that encodes definitions in the grammar (a set of rules that is used to construct computer programs – NN in this case). Then the program is executed and fitness is recorded. The genetic algorithm evolves chromosomes until an optimal solution is found, using balanced classification error as the fitness function (lower error represents higher fitness). GENN automatically selects the inputs from a pool of variables, optimizes synaptic weights, and evolves the architecture of the network, automatically selecting the appropriate network architecture for a dataset.

In the case of missing data the algorithm does not include that observation in the calculation of classification error. Only the particular missing instance is ignored, not all data for an entire individual or entire locus. Configuration parameters used in the current analyses were: 10 demes, migration every 25 generations, population size of 200 per deme, maximum of 200 generations, crossover rate of 0.9, tournament selection, standard two-point crossover, selection and a reproduction rate of 0.1[8].

### Multifactor Dimensionality Reduction (MDR)

The steps of MDR, and details of the MDR analyses presented here have been previously described[11]. Briefly, in the first step, the data is divided into a training set and an independent testing set for cross validation. Second, a set of *n *genetic and/or environmental factors are selected. These factors and their multi-factor classes are divided in *n*-dimensional space. Then the ratio of cases to controls is calculated within each multifactor class. Each multifactor cell class is then labelled "high risk" or "low risk" based on the ratio calculated, reducing the *n*-dimensional space to one dimension with two levels. The collection of these multifactor classes composes the MDR model for a particular combination of factors. For each possible model size (one-locus, two-locus, etc.) a single MDR model is chosen that has the lowest number of misclassified individuals. In order to evaluate the predictive ability of the model, prediction error is calculated using 10-fold cross-validation. The result is a set of models, one for each model size considered. From these models, a final model is chosen based on minimization of prediction error and maximization of cross validation consistency (number of times a particular set of factors is identified across the cross validation subsets).

### Data Simulations

**Figure 2:**
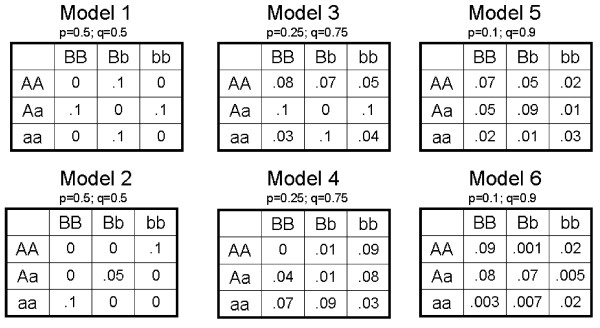
Penetrance Functions used to simulate epistasis models.

Datasets were originally generated and described for Ritchie et al, 2003[10]. Briefly, case-control data were generated under six different two-locus epistatic models, where the functional variables are single-nucleotide polymorphisms (SNPs). Data were generated using penetrance functions (shown in Figure 2), where a risk of disease is specified for each genotype combination, using software described in[13]. Each dataset is comprised of 200 cases and 200 controls, with a total of 10 biallelic SNPs each.

Each model was simulated in the presence and absence of common sources of noise, including: 5% genotyping error (GE), 5% missing data (MS), 50% phenocopy (PC), and 50% genetic heterogeneity (GH). For each model, 100 datasets were generated in the absence of any type of noise, 100 datasets were generated for each type of noise, and 100 datasets were generated for each 2, 3, or 4-way combination of the sources of noise. 96 sets of 100 datasets were generated in total.

As previously described[10], GE was simulated using a directed-error model[14] so that 5% of genotypes were biased towards one allele. MS were simulated by randomly removing 5% of genotype information. PC was simulated so that 50% of cases actually had genotypes that correspond to low-risk profiles according to the penetrance function of the corresponding epistasis model. This simulation corresponds to case status that is due to a random environmental effect. 50% GH was simulated by using a second penetrance function to define half of the cases, so that two different two-locus models predicted disease risk.

## Results

The results of all analyses were first considered under a very strict definition of power. Initially, power was defined as the proportion of times across dataset replicates the method found all functional loci, with no false positives. For the majority of models (those without GH), a two-locus model was simulated, so the correct two loci, with no false positives must be chosen as the best final model under this definition of power. For those models with GH, two different two-locus models that both independently confer disease risk were simulated. Therefore, under this strict power definition, all four functional loci must be included in the final best model, with no false positives.

The results of the MDR analyses under this definition of power, as found in[10] are shown in Table 1. GENN results using the same definition of power are shown in Table 2. Similar trends are seen for both methods. MS and GE have a very minimal effect on the power. PC decreases the power of both methods, though MDR slightly outperforms GENN in the presence of this type of noise. GH decreases power the most for both methods, which is unsurprising with this strict power definition. Even without the analytical challenge presented by two competing models, it is more difficult to detect a four-locus model than a two locus model. Additionally, PC and GH in the current simulations may also result in greater decreases in power because they account for an overall greater percentage of error in the datasets as compared to MS and GE (50% compared to 5%).

**Table 1:** Power of MDR (from Ritchie et al. 2003) to detect correct functional epistatic loci.

Source of Noise	Power (%)					
	
	Model 1	Model 2	Model 3	Model 4	Model 5	Model 6
None	100	100	99	99	82	84
GE	100	100	100	97	80	92
GH	3	41	2	3	4	4
PC	90	99	45	32	30	32
MS	100	100	99	97	82	87
GE+GH	4	41	2	3	4	6
GE+PC	94	99	41	48	28	33
GE+MS	100	100	98	98	74	84
GH+PC	0	1	0	0	0	0
GH+MS	5	38	0	2	4	6
PC+MS	96	99	42	43	14	16
GE+GH+PC	1	1	0	0	0	0
GE+GH+MS	6	34	2	1	3	7
GH+PC+MS	0	0	0	0	0	0
GE+PC+MS	94	100	48	42	18	16
GE+GH+PC+MS	0	1	0	1	0	0

GE = 5% Genotyping Error; GH = 50% Genetic Heterogeneity; PC = 50% Phenocopy; MS = 5% Missing Data

**Table 2:** Power of GENN to detect correct functional epistatic loci.

Source of Noise	Power (%)					
	
	Model 1	Model 2	Model 3	Model 4	Model 5	Model 6
None	100	100	96	91	69	72
GE	100	100	96	85	58	68
GH	7	4	15	16	14	16
PC	88	92	21	12	17	21
MS	100	100	99	82	42	74
GE+GH	3	6	14	16	11	9
GE+PC	92	95	19	11	12	16
GE+MS	100	100	93	75	48	58
GH+PC	9	9	13	15	10	11
GH+MS	1	0	0	0	0	1
PC+MS	65	85	18	13	7	9
GE+GH+PC	3	9	2	2	7	3
GE+GH+MS	5	1	0	0	0	0
GH+PC+MS	0	0	0	0	0	0
GE+PC+MS	62	81	14	9	9	9
GE+GH+PC+MS	0	0	0	0	0	0

GE = 5% Genotyping Error; GH = 50% Genetic Heterogeneity; PC = 50% Phenocopy; MS = 5% Missing Data

No synergistic effect is seen between PC and GE or MS for either method. However, different synergistic effects are seen with other combinations of error in comparing the results of the two methods. The MDR results show a major synergistic effect of PC and GH, as any combination of PC and GH, alone or with other types of error, causes the greatest decrease in power. This is unsurprising, because those datasets with only one single type of error that had the greatest drop in power were PC and GH as mentioned above. One would expect to find similar results when these two types of error are combined. The GENN results, however, do not demonstrate this effect. When PC and GH are present, the power is comparable to GH alone. However, there does seem to be a synergistic effect of GH and MS for GENN that is not seen with MDR under this strict definition of power. This effect is lessened for other definitions of power.

It is important to note that for most combinations of error, the power of GENN is generally comparable to that of MDR under this original definition of power. The evolutionary computation approach of GENN does not require an exhaustive exploration of all possible combinations of variables, as MDR does. This is an important advantage in terms of computation time; however, this advantage must not come at the cost of power.

Recognizing the extremely stringent nature of the original definition of power, especially for the models with GH, the results were re-examined. While it would be ideal for a method to identify all important signals within a dataset, it is important to know what signals a method is able to detect. To gain a more complete understanding of the performance of GENN on models with GH, several other definitions of power were considered. Again, these results are generally compared to those of MDR[15].

First, the power of each method to detect the primary genetic model (not including any loci from the second model or any false positive loci) was considered. The MDR and GENN results under this definition of power are shown in Tables 3 and 4 respectively. As these results demonstrate, the power increases greatly to detect one of the two underlying disease models than to find all four functionally loci. Also, the power of GENN is comparable, if not a little higher than that of MDR under this definition.

**Table 3:** Power of MDR (from Ritchie et al. 2007) to detect primary genetic model in data with genetic heterogeneity.

Source of Noise	Power (%) to Detect Primary Model (5,10)					
	
	Model 1	Model 2	Model 3	Model 4	Model 5	Model 6
GH	30	18	19	25	8	8
GE+GH	39	18	19	25	8	8
GH+PC	11	18	5	3	4	2
GH+MS	28	23	19	19	9	13
GE+GH+PC	10	18	8	4	3	3
GE+GH+MS	29	22	21	20	7	4
GH+PC+MS	12	22	5	5	2	4
GE+GH+PC+MS	16	17	4	3	1	3

GE = 5% Genotyping Error; GH = 50% Genetic Heterogeneity; PC = 50% Phenocopy; MS = 5% Missing Data

**Table 4:** Power of GENN to detect primary genetic model in data with genetic heterogeneity.

Source of Noise	Power (%) to Detect Primary Model (5,10)					
	
	Model 1	Model 2	Model 3	Model 4	Model 5	Model 6
GH	34	49	20	13	8	11
GE+GH	48	49	20	13	8	10
GH+PC	10	11	3	0	4	3
GH+MS	31	35	12	11	9	9
GE+GH+PC	11	8	3	0	1	2
GE+GH+MS	43	29	13	7	8	5
GH+PC+MS	3	8	3	1	5	3
GE+GH+PC+MS	4	8	1	3	0	2

GE = 5% Genotyping Error; GH = 50% Genetic Heterogeneity; PC = 50% Phenocopy; MS = 5% Missing Data

Next, the power of each method to detect either underlying genetic model was examined. The MDR and GENN results are shown in Tables 5 and 6 respectively. Again, the power increases for both methods as the definition is expanded. It is important to note that the power of GENN is generally higher than that of MDR in these results. The power of GENN under this definition is actually comparable to its power to detect these genetic models in the complete absence of error, shown in Table 2.

**Table 5:** Power of MDR (from Ritchie et al. 2007) to detect either genetic model in data with genetic heterogeneity.

Source of Noise	Power (%) to Detect Either Model (5,10 or 3,4)					
	
	Model 1	Model 2	Model 3	Model 4	Model 5	Model 6
GH	70	34	42	41	20	19
GE+GH	69	34	42	41	20	19
GH+PC	24	35	9	8	7	5
GH+MS	65	40	42	31	18	22
GE+GH+PC	27	35	10	8	7	6
GE+GH+MS	64	44	42	41	16	11
GH+PC+MS	23	38	9	10	4	6
GE+GH+PC+MS	31	36	9	7	4	3

GE = 5% Genotyping Error; GH = 50% Genetic Heterogeneity; PC = 50% Phenocopy; MS = 5% Missing Data

**Table 6:** Power of GENN to detect either genetic model in data with genetic heterogeneity.

Source of Noise	Power (%) to Detect Either Model (5,10 or 3,4)					
	
	Model 1	Model 2	Model 3	Model 4	Model 5	Model 6
GH	79	89	38	24	16	19
GE+GH	92	90	41	24	16	22
GH+PC	22	22	4	5	6	4
GH+MS	65	62	26	19	17	15
GE+GH+PC	23	23	4	3	2	4
GE+GH+MS	83	61	23	15	17	10
GH+PC+MS	13	17	3	2	7	4
GE+GH+PC+MS	7	16	2	4	1	3

GE = 5% Genotyping Error; GH = 50% Genetic Heterogeneity; PC = 50% Phenocopy; MS = 5% Missing Data

Finally, the power of each method to detect only correct loci was examined. Under this definition of power, any combination of the four total functional loci simulated as the best model is considered important. The results are shown in Tables 7 and 8. Note this is a different definition than was used in[15]. In [15], the power to detect any correct loci, regardless of false-positive loci in the model was examined. Currently, only models with no false positive loci are considered. For both methods, the power is highest for models with genetic heterogeneity under this definition of power. This is expected since this final definition is the least strict, and it is encouraging that this increase is so large compared to the original very stringent definition. The comparison between the MDR and GENN results demonstrate a substantial difference between the power results of the two methods. GENN has substantially higher power to detect only correct loci than MDR.

**Table 7:** Power of MDR to detect only correct loci (from either/both genetic models) in data with genetic heterogeneity. GE = 5% Genotyping Error; GH = 50% Genetic Heterogeneity; PC = 50% Phenocopy; MS = 5% Missing Data

Source of Noise	Power (%) to Detect Only Correct Loci					
	
	Model 1	Model 2	Model 3	Model 4	Model 5	Model 6
GH	71	36	46	45	26	21
GE+GH	71	36	46	45	26	21
GH+PC	29	39	11	8	10	7
GH+MS	68	41	48	36	21	29
GE+GH+PC	30	39	13	9	9	10
GE+GH+MS	65	46	45	45	20	16
GH+PC+MS	27	42	10	17	10	11
GE+GH+PC+MS	34	39	11	15	8	10

**Table 8:** Power of GENN to detect only correct loci (from either/both genetic models) in data with genetic heterogeneity.

Source of Noise	Power (%) to Detect Only Correct Loci					
	
	Model 1	Model 2	Model 3	Model 4	Model 5	Model 6
GH	100	100	83	71	57	68
GE+GH	100	100	83	73	58	49
GH+PC	59	57	42	49	35	40
GH+MS	99	99	77	61	46	61
GE+GH+PC	63	81	39	40	23	32
GE+GH+MS	100	100	79	72	58	53
GH+PC+MS	57	68	41	50	31	31
GE+GH+PC+MS	50	74	37	42	31	34

GE = 5% Genotyping Error; GH = 50% Genetic Heterogeneity; PC = 50% Phenocopy; MS = 5% Missing Data

## Conclusion

The results of the current study demonstrate that GENN is relatively robust to common types of noise. GENN has excellent power in the presence of GE and/or MS, but is more impacted by PC and GH. Strikingly, when the performance is compared to that of MDR, GENN has higher power to detect only true positive loci. GENN's advantage over MDR in the presence of GH may be due to the search process used (an evolutionary strategy instead of an exhaustive search), and/or specific operators (i.e. Boolean operators[16]) used in the grammar.

These results are encouraging, but it will be important to assess the performance of GENN to detect even more complex models, particularly involving GH and PC. Theoretical and empirical studies should focus on improving the overall performance, as well as evaluating GENN's relative strengths and weaknesses compared to other computational methods in the field.

## Abbreviations

GENN: Grammatical Evolution Neural Networks; MDR: Multifactor Dimensionality Reduction; GE: Genotyping error; PC: Phenocopy; MS: Missing data; GH: Genetic heterogeneity.

## Competing interests

The authors declare that they have no competing interests.

## Authors' contributions

AAM and MDR contributed to the design of the study. AAM, ACD, and TJF contributed to the data analysis. All four authors contributed to the manuscript.
